# Dressed for Sex: Red as a Female Sexual Signal in Humans

**DOI:** 10.1371/journal.pone.0034607

**Published:** 2012-04-13

**Authors:** Andrew J. Elliot, Adam D. Pazda

**Affiliations:** Department of Clinical & Social Sciences in Psychology, University of Rochester, Rochester, New York, United States of America; University of Western Australia, Australia

## Abstract

**Background:**

In many non-human primate species, a display of red by a female serves as a sexual signal to attract male conspecifics. Red is associated with sex and romance in humans, and women convey their sexual interest to men through a variety of verbal, postural, and behavioral means. In the present research, we investigate whether female red ornamentation in non-human primates has a human analog, whereby women use a behavioral display of red to signal their sexual interest to men.

**Methodology/Principal Findings:**

Three studies tested the hypothesis that women use red clothing to communicate sexual interest to men in profile pictures on dating websites. In Study 1, women who imagined being interested in casual sex were more likely to display red (but not other colors) on their anticipated web profile picture. In Study 2, women who indicated interest in casual sex were more likely to prominently display red (but not other colors) on their actual web profile picture. In Study 3, women on a website dedicated to facilitating casual sexual relationships were more likely to prominently exhibit red (but not other colors) than women on a website dedicated to facilitating marital relationships.

**Conclusions/Significance:**

These results establish a provocative parallel between women and non-human female primates in red signal coloration in the mating game. This research shows, for the first time, a functional use of color in women's sexual self-presentation, and highlights the need to extend research on color beyond physics, physiology, and preference to psychological functioning.

## Introduction

Females in many primate species, such as baboons and chimpanzees, display red on their body (e.g., chest, genitalia) near ovulation [1]–[3]. Primatologists believe that this red ornamentation is a sexual signal designed to attract mates [4], and indeed male conspecifics respond to female red with increased masturbation and copulation attempts [5], [6]. In the present research, we examine whether this female red ornamentation has an analog in humans, whereby women use a behavioral display of red to send a sexual signal to men.

Recent research suggests that red is an aphrodisiac for men viewing women. Men viewing women on a red background or in red clothing (relative to other chromatic and achromatic backgrounds and clothing) find them more attractive and sexually desirable, intend to spend more money on them, and choose to sit closer to them [7]–[8]. Perceived sexual receptivity has been shown to mediate the red-romance link; men construe the “lady in red” as more sexually receptive and this, in turn, increases their attraction to her [9]. Although this research indicates that men interpret red on a woman as a sexual signal, it is mute with regard to the accuracy of this interpretation.

Men are commonly portrayed as the initiators of sexually-oriented communication, but research shows that women are also very active, especially in the initial stages of courtship (i.e., making the first move; [10], [11]). Women convey sexual interest to men through various overt and covert means, including verbal flirtation, establishing and maintaining eye contact, provocative body posturing, suggestive dancing, and wearing revealing clothing [12]–[16]. Here we posit that women use red clothing to communicate their sexual interest to men.

Red has been used across time and culture to symbolize female sexuality in ritual, folklore, and literature [17], [18]; red means “open for business” in red-light districts, and red is the most common color of lipstick and rouge (seen by some scholars as a way to mimic natural processes of sexual excitation; [19]). These societal uses of red are posited to emerge from and extend a biologically-engrained propensity, shared with our primate relatives, to link red and sex [7], [8]. Women may exploit this red-sex link in intersexual interaction by wearing red when seeking to signal sexual receptivity.

Our research comprises three studies designed to examine women's use of red to communicate sexual interest in picture profiles on dating websites. Web dating is both mainstream and burgeoning, with approximately 20 million users per month [20], and women using these websites are typically deeply invested in catching and holding the attention of potential mates [21], [22]. As such, this arena allows for a naturalistic examination of women's real-world behavior in the mating game, a chance to observe women's use of red ornamentation essentially “in the wild.”

All of the research reported herein was approved by the University of Rochester Institutional Review Board. Participants in Study 1 were recruited via the World Wide Web and were provided modest monetary compensation for their participation; all gave informed consent and were treated in accordance with the ethical standards expressed in the Declaration of Helsinki. The pictures coded in Studies 2 and 3 were randomly selected from web profiles within the Internet's public domain. We registered for the websites to collect frequency information and compute summary data that retained the anonymity of all users; we did not subscribe to the services of any website, create any new profiles, or in any way engage in deception in conducting this research.

## Experiment 1

### Methods

Our initial, preliminary, investigation was a scenario study about women's behavior on a dating website. We examined whether women who imagined being interested in casual sex would be more likely to display red (but not other colors) on their anticipated web profile picture.

One hundred and one females participated on the world wide web in exchange for a modest cash payment. The mean age of participants was 26.9 (range  = 18–45). Participant ethnicity was as follows: 12 Asian, 6 African-American, 30 Caucasian, 3 Hispanic, 43 Indian, 3 Native-American, and 4 unspecified. Participation was restricted to self-reported heterosexual and bisexual individuals.

Participants followed a web link to gain access to the experiment. A welcome screen indicated that the experiment was about self-presentation on the internet and would consist of reading a scenario about joining a dating website, followed by completion of a brief questionnaire. Participants were randomly assigned to read either an “interested in casual sex” scenario (coded 1) or a control scenario (coded 0). The scenarios were as follows (the casual sex condition included the parenthetical information; the control condition did not):

Imagine that you (are interested in casual sex with a guy. You) decide to join a dating website because you have heard that it is a good way to find a guy (for this type of relationship). The website allows you to post one picture, and you decide to take a picture of yourself using your cell phone.

The questionnaire that followed contained items asking participants how they would pose for the picture. The item most relevant to our hypothesis was “In the picture, what color shirt would you wear?” with four response options: Red (the color of central interest), black (a highly fashionable color for adults), blue (adults' most preferred color in general), or green (the opposite of red in many well-established color models). Participants selected one of the four colors. The other items, which preceded the color item, were: “In the picture, would you wear your hair down or up?” and “In the picture, would you wear a necklace?” Given that some participants might have difficulty imagining themselves seeking a casual sexual relationship, we included items assessing how easy the scenario was for the participant to imagine, and if she thought she would ever find herself in the situation described in the scenario. Participants responded to both items on a 1 (not at all) to 9 (extremely) scale. Upon completing the questionnaire, participants were informed that the experiment was over.

### Results

Our primary analysis tested whether women in the casual sex condition were more likely to choose red than women in the control condition. We used logistic regression with condition as the predictor variable (casual sex  = 1, control  = 0) and red as the dependent variable (chosen  = 1, not chosen  = 0) to examine this central question. The analysis revealed that casual sex condition significantly predicted wearing red, B = 85, Wald χ^2^ = 3.90, *p* = 047 (Odds ratio  = 2.34); females in the casual sex condition were more likely to wear red than those in the control condition.

In ancillary analyses, we examined whether the likelihood of choosing any of the other three colors differed as a function of condition by entering each color as the dependent variable (chosen  = 1, not chosen  = 0) in logistic regression. These analyses revealed no significant differences for any other color (*p*s>.11; see Table 1). However, these ancillary logistic regressions are not independent of the primary analysis focused on red, nor from each other, because choosing one color necessarily entails not choosing any of the other colors. Accordingly, we conducted supplementary analyses to address this independence issue. Specifically, we conducted three orthogonal chi-square analyses using Helmert contrasts [23] with the following weights for red, black, blue, and green, respectively: 3, −1, −1, −1; 0, 2, −1, −1; 0, 0, 1, −1. Only the first contrast, which represented the comparison of central interest (red vs. not red), was significant, χ^2^ (1) = 4.01 *p* = .045; the remaining contrasts revealed no significant differences (*p*s>.13).

**Table 1:** Study 1: Imagined interest in casual sex predicting color on profile picture.

		Red	Black	Blue	Green
*Frequencies*	Casual sex	23	11	11	6
	Control	13	16	18	3
*Odds ratios*		2.34*	0.58	0.49	2.09

Note. *p<05.

Logistic regressions were used to test for condition differences on the non-color self-presentation items (“hair down or up,” “wear a necklace;” yes  = 1, no  = 0). These analyses yielded no significant differences (*p*s>.16). An independent samples t-test was conducted to test for condition differences on the “find self in the situation” variable; the “ease of imagining the scenario” variable was negatively skewed, so we used a Kolmogorov-Smirnov test to examine condition differences on this variable. Both analyses yielded null results (*p*s>.19). We additionally tested for possible interactions between condition and the “find self in the situation” and “ease of imagining the situation” variables; these also yielded null results (ps>.23). Finally, all of the color results reported above remained the same (i.e., significant results remained significant; non-significant results remained non-significant) when including each of the additional variables discussed in this paragraph (independently) as covariates, and all of the results reported above remained the same when including age, Caucasian/not, and Indian/not (independently) as covariates.

## Experiment 2

### Methods

Study 2 tested whether the results from Study 1 would be borne out in women's actual behavior on a dating website. Specifically, we investigated whether women who indicated an interest in casual sex would be more likely to prominently display red (but not other colors) on their web profile picture. We made no assumptions regarding the initial reason that the photograph in question was taken; we simply predicted that women interested in casual sex would be more likely to select and display a photograph of themselves in red for their profile picture.

We randomly selected female profiles from a popular dating website. Profile owners (who use screen names to guard their identity) indicate one or more types of listed relationships that they are interested in, one of which is “casual sex.” We selected 500 profiles in which “casual sex” was indicated (coded 1) and 500 profiles in which it was not (coded 0). Inclusion criteria were as follows: ages 18–35, self-reported heterosexual or bisexual, and online during past year. Profiles satisfying these criteria were displayed by the website randomly; we selected the first 500 that indicated interest in casual sex and the first 500 that did not. Profiles that contained “neck-up” photographs, colorless photographs (e.g., black and white photographs), or pictured multiple individuals were not included in the study. Two individuals, blind to hypothesis and predictor variable, used separate computer monitors to code the most prominent clothing color on each profile picture. The four colors focused on in Study 1 were coded: Red, black, blue, and green (most prominent  = 1, not most prominent  = 0). Inter-coder agreement was excellent (Kappa >.81, *p*<.001); a third coder resolved discrepancies.

### Results

Our primary analysis tested whether women indicating an interest in casual sex were more likely to prominently display red in their profile picture than women not indicating such interest. We used logistic regression with interest in casual sex as the predictor variable (casual sex  = 1, no casual sex  = 0) and prominence of red as the dependent variable (yes  = 1, no  = 0) to examine this central question. The analysis revealed that interest in casual sex significantly predicted wearing red, B = .71, Wald χ^2^ = 8.69, *p* = .003 (Odds ratio  = 2.04); females indicating interest in casual sex were more likely to wear red than those who did not indicate it.

In ancillary analyses we examined whether the likelihood of prominently displaying any of the other three colors differed as a function of condition by entering each color as the dependent variable (yes  = 1, no  = 0) in logistic regression. These analyses revealed no significant differences for any other color (*p*s>.24; see Table 2). As in Study 1, we conducted supplementary analyses to address the independence issue raised by the ancillary analyses; we used the same chi-square analyses and Helmert contrasts used in the prior study. Only the first contrast, which represented the comparison of central interest (red vs. not red), was significant, χ^2^ (1) = 8.98, *p* = .003; the remaining contrasts revealed no significant differences (*p*s>.42).

**Table 2:** Study 2: Self-reported interest in casual sex predicting color on profile picture.

		Red	Black	Blue	Green
*Frequencies*	Yes	54	169	55	21
	No	28	187	51	21
*Odds ratios*		2.04**	0.86	1.09	1.00

Note. **p<01; profile pictures in which the woman did not prominently display one of the four target colors were not coded and are not included in the table.

Most profile owners who indicated interest in casual sex also indicated interest in additional types of relationships (e.g., friendship, pen pal). As such, we reran all of the above analyses with these other types of relationships (1 =  interested, 0 =  not interested) controlled. All of the results reported above remained the same (i.e., significant results remained significant; non-significant results remained non-significant) in these analyses. Finally, all of the results reported above remained the same when including age as a covariate (ethnicity information was not available on enough profiles to warrant retrieval).

## Experiment 3

### Method

Study 3, like Study 2, focused on women's actual behavior on a dating website. Here we sought to conceptually replicate Study 2 using a different operationalization of interest in casual sex – type of dating website.

We randomly selected 500 female profiles from a website overtly dedicated to facilitating sexual relationships (coded 1); this website emphasizes casual sexual encounters, one night stands, and swinging. In contrast, we also selected 500 female profiles from a website overtly dedicated to facilitating serious, long-term relationships (coded 0); this website emphasizes love, marriage, and commitment. Inclusion criteria were comparable to Study 2, with one exception. Both websites in the current study required entering a zip code to browse profiles, which were then sorted in order of proximity to the zip code selected. We selected the New York City zip code, 10001, because of the city's ethnically diverse population. The color coding was identical to Study 2 and focused on the same colors. Inter-coder agreement was excellent (Kappa >.80, *p*<.001); a third coder resolved discrepancies.

### Results

Our primary analysis tested whether women on the sex-focused website would be more likely to prominently display red in their profile picture than women on the marriage-focused site. We used logistic regression with type of website as the predictor variable (sex-focused  = 1, marriage-focused  = 0) and prominence of red as the dependent variable (yes  = 1, no  = 0). The analysis revealed that type of website significantly predicted wearing red, B = .95, Wald χ^2^ = 10.92, *p* = .001 (Odds ratio  = 2.58); females on the sex-focused site were more likely to wear red than those on the marriage-focused site.

In ancillary analyses we examined whether the likelihood of prominently displaying any of the other three colors differed as a function of condition by entering each color as the dependent variable (yes  = 1, no  = 0) in logistic regression. These analyses revealed no significant differences for any other color (*p*s>.15; see Table 3). As in Studies 1 and 2, we conducted supplementary analyses to address the independence issue raised by the ancillary analyses; again, we used the same chi-square analyses and Helmert contrasts used in the prior studies. Only the first contrast, which represented the comparison of central interest (red vs. not red), was significant, χ^2^(1) = 11.62, *p* = .001; the remaining contrasts revealed no significant differences (*p*s>.22). Given that age had no impact on the results of Studies 1 and 2, it was not retrieved in this study; ethnicity information was not available on either website.

**Table 3:** Study 3: Type of website sex predicting color on profile picture.

		Red	Black	Blue	Green
*Frequencies*	Sex Focus	44	158	47	25
	Marriage Focus	18	167	43	16
*Odds ratios*		2.58**	0.92	1.10	1.59

Note.**p<01; profile pictures in which the woman did not prominently display one of the four target colors were not coded and are not included in the table.

## Discussion

Our results clearly link women's red displays on dating websites to their interest in sex. Red was not the most common color worn by women interested in sex; black remained most popular for these women. However, when women did display red, the odds were much (over two times) greater that they were interested in sex than not. Given that men have a well-documented hyper-readiness to impute sexual intent to women's behavior [24], [25], we hasten to add that not all women wearing red are interested in sex. Our findings are probabilistic and must be interpreted (and applied) accordingly.

The present research establishes a parallel between human and non-human female primates in their use of red to communicate sexual availability. Importantly, our research was not designed to answer the question of the deep, underlying reason behind women's use of red signal coloration. This type of “ultimate cause” question has generated considerable debate in the non-human primate literature, with many different (non-independent) answers remaining viable possibilities (e.g., red may advertise fertility, indicate fitness, and/or promote female choice by inciting male-male competition [2], [26], [27]). It is possible that there are parallels between human and non-human primates at this deeper level. For example, some theorists posit that women display more or more vivid red on their face or lips at peak fertility [7], [28]–[30], and women may be more likely to wear red at this time to enhance or exaggerate these subtle physiological processes. On the other hand, it is possible that women simply learn through observation that men are turned on by red, and intentionally choose red attire when feeling amorous and desiring sexual attention from men. Additional research is needed to begin to address the complex ultimate cause question.

A central premise of the present research is that red signal coloration is designed to communicate a message to male receivers, but we speculate that it may have two additional, ancillary effects. First, a woman's red clothing may convey to other proximate females that she is a noteworthy competitor who is actively pursuing a partner in the mating marketplace (see [31]–[33], for related arguments on female intrasexual competition in this domain). Second, a woman who dons red may, through various self-perception processes (e.g., seeing herself in a mirror, mentally reminding herself of her attire) feel more attractive or sexy, and may therefore behave in a more outgoing, proceptive manner in her intersexual interactions (see [34]–[36], for related arguments on self-perception processes in this domain). Subsequent empirical work is needed to test these intriguing possibilities. In addition, future research could examine the “second generation” question [37] of whether the type of clothing worn by the women (e.g., provocative dress versus sweatshirt) moderates the red effect documented in the present work.

In many ways, dating websites seem an ideal place to study women's sexual self-presentation. Competition for mates is intense in this arena, making women's profile choices crucial for success at attracting and keeping men's attention [15], [38], and the relative safety and control afforded by this context undoubtedly prompts more open and forthright expression [39], [40]. Nevertheless, women willing to overtly communicate their sexual interest may be relatively high in sociosexuality or extraversion. Future research is needed to test the generalizability of our findings to more reserved women, as well as women in face-to-face interaction contexts and women experiencing more ephemeral, situational sexual interest (i.e., feeling “in the mood”). Subsequent research would do well to also investigate whether the effectiveness of women's red displays in attracting mates of different types (e.g., “cads” vs. “dads;” see [41], [42]).

The present research contributes to an emerging body of work on color and psychological functioning. Studies on both competition (and competence more broadly) and sexual attraction are beginning to document that color has important, context-specific, effects on human, as well as non-human, behavior [43]–[46]. Our research herein shows, for the first time, a functional use of color in women's sexual self-presentation. In addition, our research contributes to the nascent literature on women and casual sex, a literature that seems poised for both expansion and revision (see [47], [48]).
